# MYC Overexpression Enhances Sensitivity to MEK Inhibition in Head and Neck Squamous Cell Carcinoma

**DOI:** 10.3390/ijms26020588

**Published:** 2025-01-12

**Authors:** Cuicui Yang, Xiaowu Pang, Shaolei Teng, Shamel Wilson, Xinbin Gu, Guiqin Xie

**Affiliations:** 1 Department of Oral Pathology, Howard University, 600 W Street NW, Washington, DC 20059, USA; cuicui.yang@howard.edu (C.Y.); xpang@howard.edu (X.P.); shamel.wilson@bison.howard.edu (S.W.); xgu@howard.edu (X.G.); 2 Cancer Center, Howard University, 2041 Georgia Avenue NW, Washington, DC 20059, USA; 3 Department of Biology, Howard University, 415 College St. NW, Washington, DC 20059, USA; shaolei.teng@howard.edu

**Keywords:** HNSCC, MEK inhibition, trametinib, MYC, autophagy

## Abstract

MEK inhibitors, such as trametinib, have shown therapeutic potential in head and neck squamous cell carcinoma (HNSCC). However, the factors influencing cancer cell sensitivity and resistance to MEK inhibition remain poorly understood. In our study, we observed that MEK inhibition significantly reduced the expression of MYC, a transcription factor critical for the therapeutic response. MYC overexpression markedly enhanced the sensitivity of HNSCC cells to trametinib, as evidenced by delayed wound healing and reduced colony formation. Cell cycle analysis revealed that trametinib induced a G1 phase arrest, whereas MYC overexpression accelerated cell cycle progression, with a reduced induction of p27 and p21 and diminished decreases in E2F1 and phospho-Ser2/5 levels. Flow cytometry and protein analyses demonstrated that MYC overexpression amplified trametinib-induced apoptosis and DNA damage, as evidenced by elevated levels of pro-apoptotic markers (p53, cleaved PARP, and BIM) and γH2AX. In vivo xenograft models confirmed these findings, showing increased sensitivity to trametinib in MYC-overexpressing tumors. Moreover, MEK inhibition increased autophagy in HNSCC cells, a factor critical for therapeutic resistance. Inhibiting trametinib-induced autophagy further enhanced apoptotic cell death. These findings suggest that MYC expression and autophagy play crucial roles in HNSCC’s response to MEK inhibition. Combining trametinib with autophagy inhibition may improve therapeutic outcomes in HNSCC.

## 1. Introduction

Head and neck cancer accounts for about 4% of all cancers in the United States. More than 90% of head and neck cancers are squamous cell carcinomas (HNSCC) [1], originating from the mucosal epithelial cells lining the oral cavity, pharynx, larynx, and sinonasal tract. Standardized treatment options for HNSCC, including surgery, radiation, and chemotherapy, often fail to achieve durable effects in the majority of patients and are associated with severe side effects. Newly developed treatment modalities, such as immunotherapy, have not yielded ideal responses in HNSCC, with an overall response rate of less than 20% of the patients, regardless of their human papillomavirus (HPV) status [2,3]. Despite these efforts, the 5-year survival rate of HNSCC remains between 55 and 65% [1]. The prognosis for patients with recurrent or metastatic HNSCC patients is even poorer, with a survival rate of less than 1 year [4]. Moreover, the occurrence of HNSCC is on an upward trend, with projections indicating a potential 30% growth rate by 2030 (Global Cancer Observatory (GLOBOCAN)) [5,6]. These challenges underscore the importance of research focused on understanding the mechanisms underlying HNSCC pathogenesis and treatment resistance.

Extensive research has revealed abnormal activation of signaling pathways in HNSCC tumors. For example, overexpression of the epidermal growth factor receptor (EGFR), a membrane-bound tyrosine kinase receptor, is one of the most notable characteristics of HNSCC [1]. However, molecular targeting of EGFR with monoclonal antibodies such as cetuximab, a Food and Drug Administration (FDA)-approved strategy in HNSCC, has only yielded a moderate patient response rate, ranging from 10 to 30% when used as a monotherapy [7,8,9]. This limited efficacy is often associated with primary and/or acquired drug resistance, highlighting the need to search for alternative therapeutic targets. As a key intracellular downstream effector of EGFR signaling, the mitogen-activated protein kinase (MAPK) pathway serves as a crucial signaling hub that integrates extracellular signals to regulate various cellular processes, including cell proliferation, survival, differentiation, senescence, and drug resistance. The pathway involves a cascade of kinases, including the RASs (H/K/NRAS), RAFs (A-/B-/C-RAF), MEKs (MEK1/2), MAPKs [MAPK1 (ERK2), and MAPK3 (ERK1)], among others [10]. Based on analyses of The Cancer Genome Atlas (TCGA) dataset, it is reported that MAPK pathway mutations occur in approximately 18% of HNSCC patient tumors and that these mutations are largely activating [11]. Indeed, in recent years, new classes of MEK/MAPK inhibitors with improved potencies have been developed, showing clinical promise in various types of cancers, including HNSCC. Among these, trametinib (also known as GSK1120212) stands out as a promising MEK1/2 inhibitor for HNSCC, effectively inhibiting MEK1 and MEK2 kinases, preventing RAF-dependent MEK phosphorylation, and resulting in prolonged pERK1/2 inhibition [12]. Trametinib has received FDA approval for treating patients with advanced melanoma, thyroid cancer, and metastatic non-small cell lung cancer harboring a BRAF mutation [13,14]. Although mutations of the *BRAF* gene in HNSCC are rare [15], a phase II window-of-opportunity trial demonstrated a significant reduction in RAS/MEK/ERK pathway activation, along with notable clinical and metabolic tumor responses to trametinib in patients with oral cavity HNSCC [16]. In our preclinical study in HNSCC, we also reported that trametinib’s inhibition of MEK signaling may target EGFR and its downstream proteins [17]. While both preclinical and clinical evidence demonstrate trametinib’s potential in treating HNSCC, the molecular mechanisms underlying the vulnerability of HNSCC to trametinib remain to be elucidated.

The nuclear transcription factor c-Myc (MYC) is a crucial integrator of growth-regulatory and oncogenic signaling pathways, including the RAS-MAPK pathway [18]. MYC is well-known for its role in controlling cell cycle progression, proliferation, and apoptosis. Overexpression of MYC induces tumorigenesis, and this oncoprotein is functionally involved in a significant proportion of human cancers [19,20,21], making it an attractive target for cancer therapy. In HNSCC, high MYC expression in tumors originating from the nasopharynx is associated with shorter overall survival [22]. Importantly, recent reports indicate that MYC overexpression in HNSCC is associated with poor prognoses [23,24]. A case report identified MYC amplification as a potential mechanism of resistance to immunotherapy and suggested its use as a predictive biomarker in recurrent/metastatic HNSCC [25]. However, it is less well understood whether and how MYC affects the vulnerability of HNSCC to MEK inhibition by trametinib. In this study, we evaluated the impact of MYC on HNSCC’s response to trametinib, specifically exploring the hypersensitivity conferred by MYC overexpression to trametinib in HNSCC cells. To investigate the mechanisms underlying this hypersensitivity, we assessed the response of HNSCC cells and xenograft tumors with MYC overexpression to trametinib. Our study revealed that trametinib reduced MYC expression and increased autophagy, both of which are critical for the treatment response. MYC overexpression enhanced HNSCC cell sensitivity to trametinib, promoting apoptosis, DNA damage, cell cycle progression, and tumor growth inhibition. Inhibiting autophagy induced by trametinib further increased apoptosis. These findings suggest that MYC and autophagy are critical in modulating HNSCC’s response to MEK inhibition and that combining trametinib with autophagy inhibitors may improve therapeutic outcomes.

## 2. Results

### 2.1. Trametinib-Induced Multiple Anti-Cancer Effects in HNSCC Cells Through MEK Inhibition

Based on our previous findings demonstrating trametinib’s efficacy in suppressing HNSCC progression [17], we further investigated molecular alterations associated with the MEK pathway underlying these responses. HNSCC cells were treated with trametinib at 200 nM.

**Figure 1:**
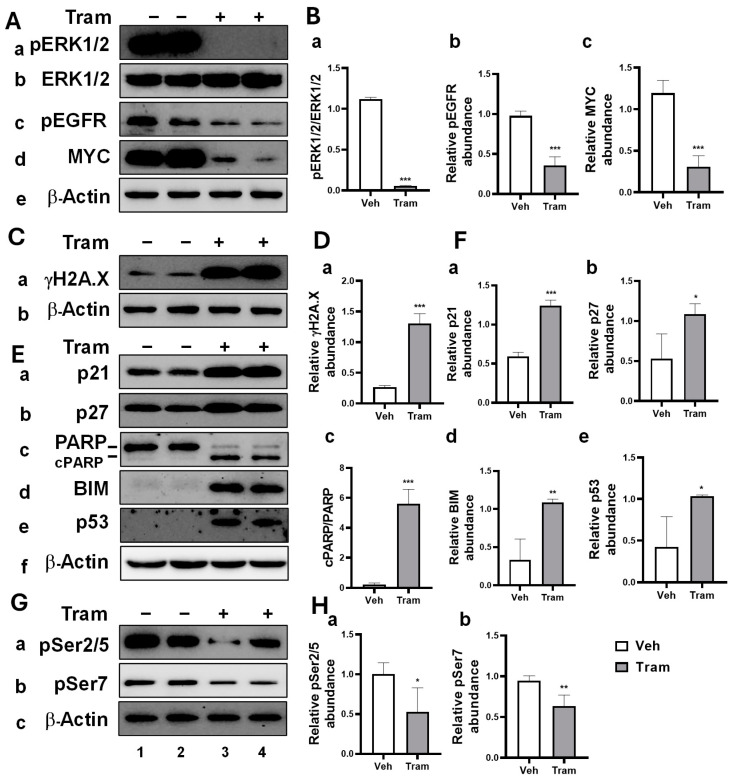
Multiple anti-cancer effects induced by trametinib through MEK/MAPK inhibition in JHU11 HNSCC cells. Total protein extracts were prepared from JHU11 cells treated with either vehicle or 200 nM trametinib for 48 h. Western blot analysis was performed to detect (**A**) pERK1/2 (**a**), total ERK1/2 (**b**), pEGFR (**c**), MYC (**d**), and β-Actin (**e**). (**B**) Protein band intensities of (**a**) pERK/ERK, (**b**) pEGFR, and (**c**) MYC were quantified and normalized to the corresponding β-actin to compare vehicle- and trametinib-treated cells. (**C**,**D**) γH2A.X (**a**) and β-Actin (**Cb**); (**E**,**F**) p21 (**a**), p27 (**b**), cPARP/PARP (**c**), BIM (**d**), p53 (**e**), and β-Actin (**Ef**); (**G**,**H**) pSer2/5 (**a**), pSer7 (**b**), and β-Actin (**Gc**). Protein band intensities for the ratio of cPARP/PARP, pEGFR, MYC, γH2A.X, p21, p27, BIM, p53, pSer2/5, and pSer7 were normalized to β-actin correspondingly and compared between vehicle-treated and trametinib-treated cells. Data from independent experiments are presented as mean ± SD (n = 3~4). Statistical significance is indicated as follows: * *p* < 0.05, ** *p* < 0.01, *** *p* < 0.001 compared to vehicle-treated controls. Abbreviations: Veh: Vehicle, Tram: Trametinib.

First, we validated trametinib’s effectiveness in inhibiting MEK signaling by assessing the ratio of phosphorylated ERK1/2 (pERK1/2) to total ERK1/2. This ratio was significantly reduced in trametinib-treated JHU11 (Figure 1(Aa,Ab,Ba)), JHU13 (Supplementary Figure S1(Aa,Ab,Ba)), and JHU22 (Supplementary Figure S2(Aa,Ab,Ba)) cells. In line with our previous findings [17], trametinib also significantly reduced EGFR activation across all three cell lines. Compared to vehicle-treated controls, the relative abundance of phosphorylated EGFR (pEGFR) was significantly decreased in JHU11 (Figure 1(Ac,Bb)), JHU13 (Supplementary Figure S1(Ac,Bb)), and JHU22 (Supplementary Figure S2(Ac,Bb)) cells. These results highlight trametinib’s ability to downregulate EGFR signaling, a critical pathway frequently overexpressed in HNSCC and known to drive cell proliferation. Moreover, trametinib treatment consistently and significantly reduced MYC protein levels in the HNSCC cells tested. This reduction was observed in JHU11 (Figure 1(Ad,Bc)), JHU13 (Supplementary Figure S1(Ad,Bc)), and JHU22 (Supplementary Figure S2(Ad,Bc)) cells compared to controls. These findings suggest that trametinib impacted MYC-regulated processes, including cell proliferation and growth inhibition.

The formation of γH2A.X, through phosphorylation at the Ser-139 residue on the histone variant H2A.X, is an early cellular response to DNA double-strand break inductions [26]. We observed that γH2A.X levels were significantly elevated in trametinib-treated cells, including JHU11 (Figure 1(Ca,Da)), JHU13 (Supplementary Figure S1(Ca,Da)), and JHU22 (Supplementary Figure S2(Ca,Da)) cells, respectively, suggesting that trametinib induced additional cytotoxicity by promoting DNA damage.

To further elucidate the molecular changes responsible for the cell cycle arrest and apoptosis previously identified via flow cytometry, we performed a Western blot analysis to examine key regulatory proteins. Cyclin-dependent kinase inhibitors, p21 (Cip1/Waf1) and p27 (Kip1), are critical regulators of the cell cycle. We observed significant increases in both p21 and p27 levels in JHU11 (Figure 1(Ea,Eb,Fa,Fb)), JHU13 (Supplementary Figure S1(Ea,Eb,Fa,Fb)), and JHU22 (Supplementary Figure S2(Ea,Eb,Fa,Fb)) cells after trametinib treatment. Apoptotic markers, such as cleaved poly (ADP-ribose) polymerase (PARP) and Bcl-2-interacting mediator of cell death (BIM), were also elevated following trametinib treatment. Specifically, the cPARP/PARP ratio and BIM levels were significantly increased in JHU11 (Figure 1(Ec,Ed,Fc,Fd)), JHU13 (Supplementary Figure S1(Ec,Ed,Fc,Fd)), and JHU22 (Supplementary Figure S2(Ec,Ed,Fc,Fd)) cells. In addition, the tumor suppressor protein p53 was upregulated as well in JHU11 (Figure 1(Ee,Fe)), JHU13 (Supplementary Figure S1(Ee,Fe)), and JHU22 (Supplementary Figure S2(Ee,Fe)) cells compared to vehicle controls, respectively.

Given the importance of RNAP II carboxy-terminal domain (CTD) phosphorylation in transcriptional regulation, we assessed whether trametinib affected RNAP II CTD phosphorylation. After 48 h of treatment, we observed a significant reduction in RNAP II CTD phosphorylation at the pSer2/5 and pSer7 sites compared to vehicle controls. Specifically, phosphorylation levels of pSer2/5 and pSer7 were decreased in JHU11 (Figure 1(Ga,Gb,Ha,Hb)), JHU13 (Supplementary Figure S1(Ga,Gb,Ha,Hb)), and JHU22 (Supplementary Figure S2(Ga,Gb,Ha,Hb)) cells. These findings suggest that trametinib-induced MEK inhibition exerted potent anti-cancer effects in HNSCC by targeting key regulators of cancer survival, apoptosis, DNA damage responses, and gene transcription. MEK inhibition also significantly affected the expression of critical transcription factors like MYC, raising the possibility that MYC may influence the sensitivity of cancer cells to MEK inhibition as it regulates the expression of numerous genes.

### 2.2. MYC Overexpression Renders HNSCC Cells More Sensitive to Trametinib

**Figure 2:**
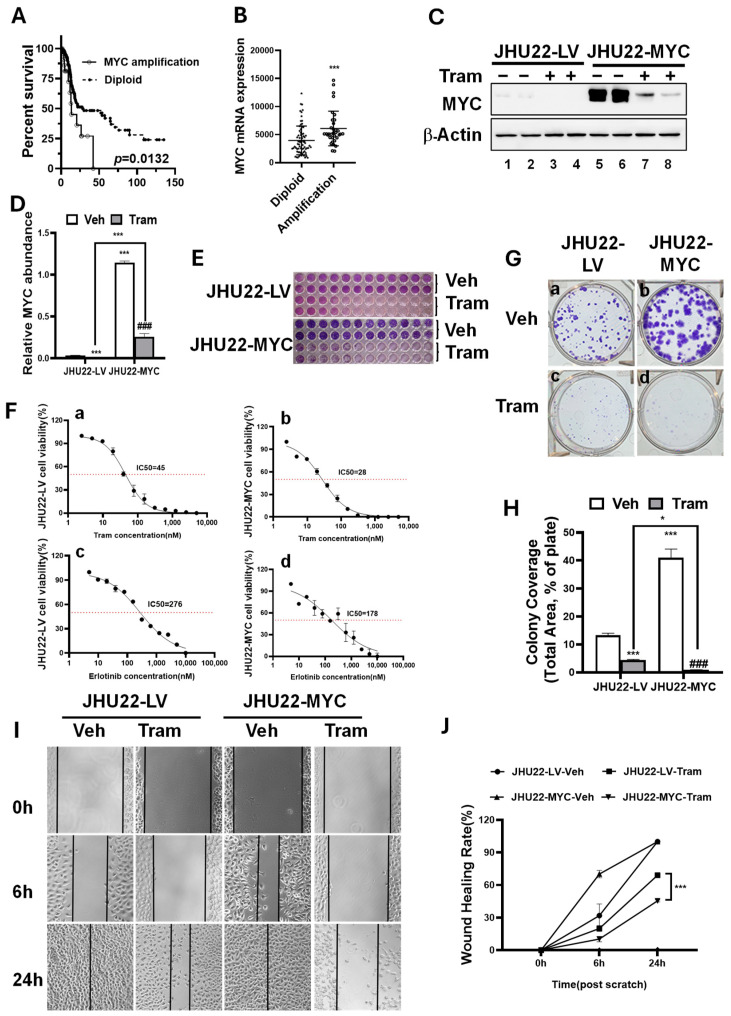
MYC overexpression renders JHU22 HNSCC cells more sensitive to trametinib. (**A**) Overall survival percentage of HNSCC patients with (solid line) versus without MYC amplification (dotted line) (*p* = 0.013). (**B**) Increased MYC mRNA expression is observed in HNSCC patients with MYC amplification. All data are from the TCGA database (279 samples), Nature 2015, accessed via cBioPortal.org [27,28]. (**C**) Total protein extracts were obtained from JHU22-LV and JHU22-MYC cells treated with either vehicle or 200 nM trametinib for 48 h. Western blot analysis was performed to detect MYC and β-Actin. (**D**) MYC band intensities were quantified and normalized to the corresponding β-actin to compare vehicle- and trametinib-treated cells (n = 4). (**E**) A dose-escalation study was conducted to evaluate the effects of increasing concentrations of trametinib on cell viability in JHU22-LV and JHU22-MYC cells, with treatment lasting for 7 days. Images represent two replicates. (**F**) IC50 values for trametinib (**a**,**b**) and erlotinib (**c**,**d**) were determined in JHU22-LV and JHU22-MYC cells, respectively. The red dotted line indicates 50% viability, corresponding to the IC50 value. (**G**) Colony formation assays were performed to assess the proliferative ability of JHU22-LV (**a**,**c**) and JHU22-MYC (**b**,**d**) cells following treatment with either vehicle or trametinib (200 nM) for 7 days. (**H**) Bar graphs and statistical analysis of the colony coverage (Total area, % of plate). (**I**) Wound healing assays were used to examine changes in migratory capacity between JHU22-LV and JHU22-MYC cells, with treatments of either vehicle or trametinib (200 nM) conducted at 0, 6, and 24 h. (**J**) Line graphs and statistical analysis of wound healing assays. Data from independent experiments are presented as mean ± SD (n = 4). Statistical significance is indicated as follows: * *p* < 0.05; *** *p* < 0.001 compared to JHU22-LV vehicle-treated controls. ### *p* < 0.001 compared to JHU22-MYC vehicle-treated controls. Abbreviations: Veh: Vehicle, Tram: Trametinib.

MYC plays a critical role in driving tumorigenesis across multiple cancer types. Given its role as a key target of MEK inhibition in various HNSCC cells, exploring how MYC influences the sensitivity of cancer cells to MEK inhibition is particularly intriguing. To assess the impact of MYC amplification on patient prognosis in HNSCC, we reanalyzed The Cancer Genome Atlas (TCGA) dataset. In silico analysis of MYC in the TCGA HNSCC dataset (n = 279, Nature 2015) was conducted using data obtained from the cBioPortal [27,28] for Cancer Genomics (http://cbioportal.org, URL accessed on 8 September 2024). Our analysis showed that MYC mRNA expression was significantly higher in patients with *MYC* amplification compared to those without, highlighting MYC as one of the frequently amplified genes in HNSCC (Figure 2B). Importantly, MYC amplification was significantly correlated with shorter overall survival (OS) in HNSCC patients (Figure 2A), reinforcing the association between MYC overexpression and poor prognosis, as reported in recent studies [23,24].

The impact of MYC-driven oncogenic alterations on the efficacy of trametinib against HNSCC remains understudied. To investigate how MYC influences the response of HNSCC to MEK inhibition by trametinib, we overexpressed MYC protein in JHU22 cells. As anticipated, JHU22 cells with MYC overexpression (designated as JHU22-MYC) exhibited significantly elevated MYC protein levels compared to control cells infected with a mock lentivirus (designated as JHU22-LV) (Figure 2C, bands 5 and 6 vs. 1 and 2; Figure 2D). We then assessed the effects of trametinib on cell proliferation in both JHU22-LV and JHU22-MYC cells. As shown in Figure 2E, trametinib reduced cell viability in a dose-dependent manner, with IC50 values of 45 nM for JHU22-LV cells (Figure 2(Fa)) and 28 nM for JHU22-MYC cells (Figure 2(Fb)). These results indicate that MYC overexpression may enhance the sensitivity of JHU22 cells to trametinib, raising the question whether MYC overexpression could also influence the response to other therapeutic modalities. To explore this further, we assessed the sensitivity of JHU22-LV and JHU22-MYC cells to erlotinib, a tyrosine kinase inhibitor targeting the EGFR pathway. Erlotinib reduced cell viability in a dose-dependent manner, with IC50 values of 276 nM for JHU22-LV cells (Figure 2(Fc)) and 178 nM for JHU22-MYC cells (Figure 2(Fd)). These results suggest that MYC overexpression in HNSCC cells may enhance drug sensitivity and indicate a potential dependence on this oncogene.

Next, we investigated whether MYC overexpression affects the trametinib-induced inhibition of long-term proliferation and migration in JHU22 cells. JHU22-LV and JHU22-MYC cells were treated with either a vehicle or trametinib (200 nM) for 5 days. Long-term proliferation and survival were then evaluated through colony formation assays. Trametinib treatment reduced colony formation in both cell lines, with the percentage of positive areas decreasing from 13.3% to 4.4% in JHU22-LV cells (Figure 2(Ga) vs. Figure 2(Gc); Figure 2H) and from 41% to 0.8% in JHU22-MYC cells (Figure 2(Gb) vs. Figure 2(Gd); Figure 2H) compared to vehicle-treated controls, indicating while MYC overexpression increased the proliferation ability of colony formation in JHU22-MYC cells, trametinib induced a stronger inhibition of proliferation in these MYC-overexpressing cells. Additionally, to assess the impact of MYC overexpression on cell migration, we performed wound healing assays over a 24 h period, with evaluations at 0, 6, and 24 h following treatment with either a vehicle or trametinib. Trametinib treatment significantly slowed cell migration compared to vehicle treatment, with the most pronounced effect observed at the 24 h time point, particularly in JHU22-MYC cells (Figure 2I,J). This suggests that MYC overexpression sensitizes cells to trametinib’s anti-migratory effects. Thus, we demonstrated that MYC overexpression enhanced the sensitivity of JHU22-MYC cells to trametinib, amplifying its inhibitory effects on both cell proliferation and migration. This suggests that MYC overexpression may enhance the sensitivity of HNSCC to the anti-cancer effects of trametinib.

### 2.3. MYC Overexpression Alters the Response of HNSCC Cells to Trametinib

**Figure 3:**
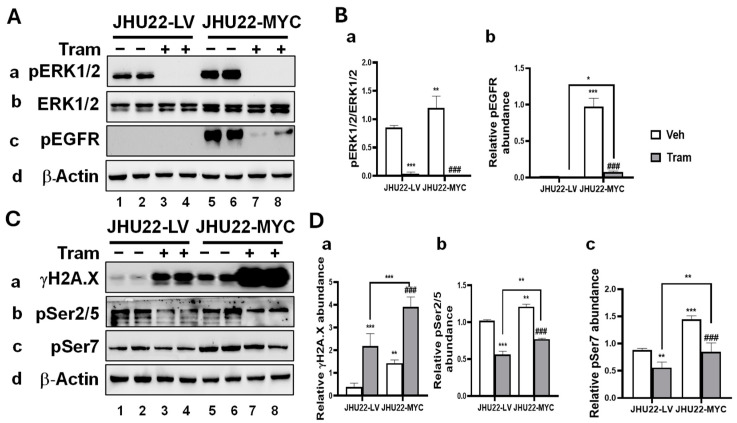
MYC overexpression alters the molecular response of HNSCC cells to trametinib. Total protein extracts were prepared from JHU22-LV and JHU22-MYC cells treated with either vehicle or trametinib (200 nM) for 48 h. (**A**) Western blot analysis was performed to detect pERK1/2 (**a**), total ERK1/2 (**b**), pEGFR (**c**) and β-Actin (**d**). (**B**) The pERK1/2/ERK1/2 ratio (**a**) or protein band intensity of pEGFR (**b**) was quantified and normalized to the corresponding β-actin to compare the effects between vehicle-treated and trametinib-treated cells (n = 4). (**C**) Western blot analysis was also conducted to detect γH2A.X (**a**), pSer2/5 (**b**), pSer7 (**c**), and β-actin (**d**) in JHU22-LV and JHU22-MYC cells treated with vehicle or trametinib for 48 h. (**D**) The protein band intensities of γH2A.X (**a**), pSer2/5 (**b**), and pSer7 (**c**) were quantified and normalized to the corresponding β-actin to compare the effects between vehicle-treated and trametinib-treated cells. Data from four independent experiments are presented as mean ± SD (n = 4). Statistical significance is indicated as follows: * *p* < 0.05; ** *p* < 0.01; *** *p* < 0.001 compared to JHU22-LV vehicle-treated controls. ### *p* < 0.001 compared to JHU22-MYC vehicle-treated controls. Abbreviations: Veh: Vehicle, Tram: Trametinib.

Given trametinib’s role in regulating critical cellular processes such as proliferation, transcription, and DNA damage responses in HNSCC cells, we explored how MYC overexpression affects these molecular alterations. Trametinib effectively diminished the pERK/ERK ratio in both JHU22-LV and JHU22-MYC cells compared to vehicle-treated controls (Figure 3(Aa,Ab,Ba)), confirming the successful inhibition of the ERK signaling pathway, even in the presence of MYC overexpression. MYC overexpression greatly increased the relative abundance of pEGFR in JHU22-MYC cells compared with JHU22-LV cells (Figure 3(Ac), bands 5 and 6 vs. bands 1 and 2). However, treatment with trametinib led to a marked reduction in pEGFR levels in JHU22-MYC cells (Figure 3(Ac), bands 7 and 8 vs. bands 5 and 6; Figure 3(Bb)). As shown in Figure 2C,D, compared with vehicle treatment, trametinib significantly decreased MYC protein levels in both JHU22-LV cells (Figure 2C, bands 3 and 4 vs. bands 1 and 2) and JHU22-MYC cells (Figure 2C, bands 7 and 8 vs. bands 5 and 6). While the persistence of MYC in JHU22-MYC cells likely reflects its lentiviral overexpression, the significant reduction indicates that trametinib can still successfully downregulate MYC protein levels. These findings underscore trametinib’s ability to inhibit key signaling pathways involved in cell proliferation and survival in HNSCC cells, even in the context of MYC overexpression.

We next assessed the DNA damage marker γH2A.X. We found that MYC overexpression significantly increased γH2A.X abundance (Figure 3(Ca), bands 5 and 6 vs. bands 1 and 2), and treatment with trametinib further elevated γH2A.X levels in JHU22-MYC cells compared to vehicle treatment (Figure 3(Ca), bands 7 and 8 vs. bands 5 and 6). Additionally, the trametinib-induced increase in γH2A.X was more pronounced in JHU22-MYC cells compared to JHU22-LV cells (Figure 3(Ca), bands 7 and 8 vs. bands 3 and 4; Figure 3(Da)), suggesting a significantly enhanced DNA damage response in these cells.

To explore whether MYC overexpression influences trametinib-induced transcriptional changes, we examined the phosphorylation status of RNAP II CTD after 48 h of treatment. MYC overexpression significantly increased the abundance of both pSer2/5 (Figure 3(Cb), bands 5 and 6 vs. bands 1 and 2; Figure 3(Db)) and pSer7 (Figure 3(Cc), bands 5 and 6 vs. bands 1 and 2; Figure 3(Dc)), indicating increased transcriptional activity in JHU22-MYC cells compared to JHU22-LV cells. Moreover, compared to their respective vehicle-treated controls, trametinib treatment in JHU22-LV or JHU-MYC cells led to a significant decrease in phosphorylation at both pSer2/5 and pSer7 (Figure 3(Cb,Cc,Db,Dc)). However, the trametinib-induced decrease in pSer2/5 (Figure 3(Cb), bands 7 and 8 vs. bands 3 and 4; Figure 3(Db)) or pSer7 (Figure 3(Cc), bands 7 and 8 vs. bands 3 and 4; Figure 3(Dc)) was less pronounced in JHU22-MYC cells compared to JHU22-LV cells. These findings suggest that MYC overexpression may partially counteract trametinib-induced reductions in RNAP II CTD phosphorylation, potentially impacting transcriptional activity in response to the treatment.

Together, we demonstrated that MYC overexpression modified the cellular response to trametinib by enhancing the DNA damage response, as evidenced by the increased γH2A.X levels, while also partially mitigating transcriptional inhibition in HNSCC cells.

### 2.4. MYC Overexpression Promotes Cell Cycle Progression and Apoptosis in Responses to Trametinib in JHU22 HNSCC Cells

**Figure 4:**
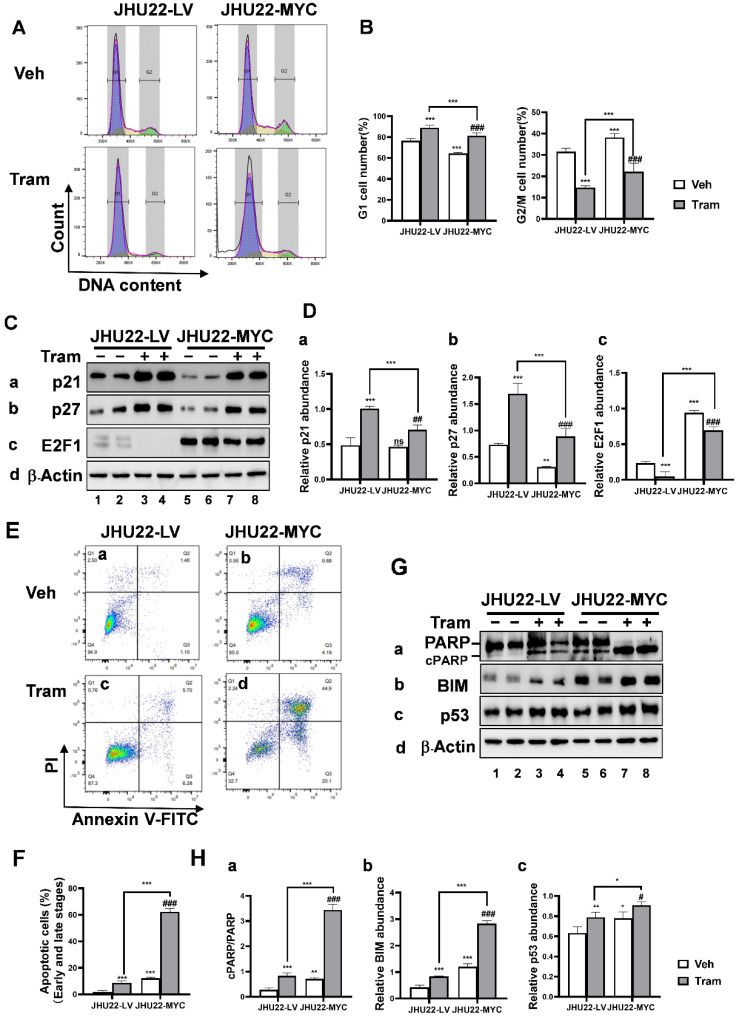
MYC overexpression in JHU22 HNSCC cells promotes cell cycle progression and apoptotic cell death in response to trametinib. (**A**) JHU22-LV and JHU22-MYC cells were treated with either vehicle or trametinib (200 nM) for 48 h, and flow cytometric analysis of cell cycle phases was performed following DAPI staining. Blue: G1; Yellow: S Phase; Green: G2/M; Magenta: Sum. (**B**) The percentage of cells in the G1 or G2/M phase in both JHU22-LV and JHU22-MYC cells was quantified (n = 4). (**C**) Total protein extracts were obtained from JHU22-LV and JHU22-MYC cells following treatment with either vehicle or trametinib (200 nM) for 48 h. Western blot analysis was conducted to detect p21 (**a**), p27 (**b**), E2F1 (**c**), and β-actin (**d**). (**D**) Protein band intensities of p21 (**a**), p27 (**b**), and E2F1 (**c**) were quantified and normalized to the corresponding β-actin to compare the effects between vehicle-treated and trametinib-treated cells (n = 4). (**E**) JHU22-LV (**a**,**c**) and JHU22-MYC (**b**,**d**) cells were treated with either vehicle or trametinib (200 nM) for 48 h and then collected for annexin V and PI staining, followed by flow cytometric analysis to assess apoptotic cells. Pseudo color plots are used to indicate cell density or number. (**F**) The populations of apoptotic cells in both early and late stages were quantified (n = 4). (**G**) Western blot analysis was performed to detect cPARP/PARP (**a**), BIM (**b**), p53 (**c**), and β-actin (**d**) in JHU22-LV and JHU22-MYC cells. (**H**) The cPARP/PARP ratio (**a**) or protein band intensities of BIM (**b**) and p53 (**c**) were quantified relative to the corresponding β-actin to compare the effects between vehicle-treated and trametinib-treated cells. Data from four independent experiments are presented as mean ± SD (n = 4). Statistical significance is indicated as follows: * *p* < 0.05; ** *p* < 0.01; *** *p* < 0.001 compared to JHU22-LV vehicle-treated controls. # *p* < 0.05; ## *p* < 0.01; ### *p* < 0.001 compared to JHU22-MYC vehicle-treated controls. ns: not significant. Abbreviations: Veh: Vehicle, Tram: Trametinib.

JHU22-LV and JHU22-MYC cells were treated with either a vehicle or trametinib (200 nM) for 48 h, followed by DAPI staining and flow cytometry analysis to evaluate cell cycle phases. Compared to JHU22-LV cells, MYC overexpression in JHU22-MYC cells significantly decreased the proportion of cells in the G1 phase and increased the proportion of cells in the G2/M phase (Figure 4A, upper panels, and Figure 4B), indicating enhanced cell cycle progression. When treated with trametinib, both JHU22-LV and JHU22-MYC cells showed greater accumulation in the G1 phase compared to their respective vehicle-treated controls, along with a significant decrease in the percentage of cells in the G2/M phase (Figure 4A, upper vs. lower panels, and Figure 4B). Notably, in response to trametinib, JHU22-MYC cells exhibited significantly fewer cells in the G1 phase and more cells in the G2/M phase compared to JHU22-LV cells (Figure 4A, lower panels, and Figure 4B), suggesting that MYC overexpression significantly reduced trametinib’s effectiveness in inducing G1 arrest.

Among cyclin-dependent kinase inhibitors, p21 primarily induces G1-phase arrest, while p27 controls progression from the G1 to the S phase. In Western blotting analysis, MYC overexpression showed a trend toward decreased abundance of p27 (Figure 4(Cb), bands 5 and 6 vs. bands 1 and 2; Figure 4(Db)) but had no significant effect on p21 levels (Figure 4(Ca), bands 5 and 6 vs. bands 1 and 2; Figure 4(Da)). Trametinib treatment significantly increased p21 as well as p27 levels in both JHU22-LV and JHU22-MYC cells compared to their respective vehicle-treated controls. However, this increase in p21 (Figure 4(Ca), bands 7 and 8 vs. bands 5 and 6; Figure 4(Da)) and p27 (Figure 4(Cb), bands 7 and 8 vs. bands 5 and 6; Figure 4(Db)) induced by trametinib was smaller in MYC-overexpressing cells than in JHU22-LV cells. This diminished upregulation of p21 and p27 correlated with the reduced G1-phase arrest observed, indicating the attenuated increase in these inhibitors may contribute to the impaired G1-phase arrest in JHU22-MYC cells. E2F1 transcription factors, crucial regulators of the cell cycle—particularly in the transition from the G1 to the S phase and progression through the G2/M phases—were also affected. MYC overexpression significantly increased E2F1 expression in JHU22-MYC cells compared to JHU22-LV cells (Figure 4(Cc), bands 5 and 6 vs. bands 1 and 2; Figure 4(Dc)). After trametinib treatment, residual E2F1 expression remained apparent in JHU22-MYC cells, in contrast to the markedly reduced E2F1 expression in JHU22-LV cells (Figure 4(Cc), bands 7 and 8 vs. bands 3 and 4). The persistent E2F1 expression in JHU22-MYC cells, even after trametinib treatment, likely also contributed to the reduced G1-phase arrest and increased transition to the G2/M phase. These findings indicate that MYC overexpression may modify the response to trametinib in JHU22-MYC cells by disrupting the G1-phase arrest and promoting the proportion of cells transitioning to the G2/M phase, thereby reducing trametinib’s overall efficacy in inducing cell cycle arrest. The observed changes in p21 and p27 levels, along with the sustained presence of E2F1 in MYC-overexpressing cells, highlight a deregulated cell cycle that may undermine the drug’s impact on both G1 and G2/M phases.

Next, we examined the impact of MYC overexpression on apoptotic cell death. JHU22-LV and JHU22-MYC cells were treated with either a vehicle or trametinib (200 nM) for 48 h and analyzed for apoptosis using annexin V and PI (propidium iodide) staining. MYC overexpression significantly increased the proportion of apoptotic cells at both early and late stages in JHU22-MYC cells compared to that in JHU22-LV cells (Figure 4(Eb) vs. Figure 4(Ea); Figure 4F), indicating enhanced cell apoptosis. Trametinib treatment further induced the percentage of apoptotic cells (early and late stages) from 1.9% to 8.7% in JHU22-LV cells and from 12.2% to 62.4% in JHU22-MYC cells compared to their respective vehicle control. This increase was reflected by a higher proportion of annexin V-positive cells, indicating greater sensitivity to apoptosis in MYC-overexpressing cells (Figure 4(Ed) vs. Figure 4(Ec); Figure 4F) in response to trametinib treatment.

A Western blot analysis revealed significant increases in pro-apoptotic proteins, including cleaved PARP (cPARP) relative to PARP (Figure 4(Ga), bands 5 and 6 vs. bands 1 and 2), BIM (Figure 4(Gb), bands 5 and 6 vs. bands 1 and 2) and p53 (Figure 4(Gc), bands 5 and 6 vs. bands 1 and 2) in JHU22-MYC cells compared to JHU22-LV cells, even in the absence of trametinib treatment. This suggests that MYC overexpression contributes, at least in part, to increased apoptotic cell death in JHU22-MYC cells, as observed in the flow cytometry analysis. Upon trametinib treatment, these proteins were further elevated in both cell types, including cPARP relative to PARP (Figure 4(Ga,Ha)), BIM (Figure 4(Gb,Hb)), and p53 (Figure 4(Gc,Hc)), compared to their respective vehicle-treated control groups. Notably, trametinib treatment rendered a greater increase in the abundance of cPARP relative to PARP (Figure 4(Ga), bands 7 and 8 vs. bands 3 and 4), BIM (Figure 4(Gb), bands 7 and 8 vs. bands 3 and 4), and p53 (Figure 4(Gc), bands 7 and 8 vs. bands 3 and 4) in JHU22-MYC cells compared to JHU22-LV cells, reinforcing the observation of higher apoptosis in JHU22-MYC cells. These results support the enhanced apoptosis observed in JHU22-MYC cells in flow cytometry.

Overall, these findings demonstrated that MYC overexpression in JHU22 HNSCC cells significantly reduced the trametinib-induced cell cycle arrest, thereby promoting cell cycle progression while enhancing the apoptotic response to trametinib treatment.

### 2.5. Inhibition of Trametinib-Induced Autophagy Enhances Apoptotic Cell Death in HNSCC Cells

**Figure 5:**
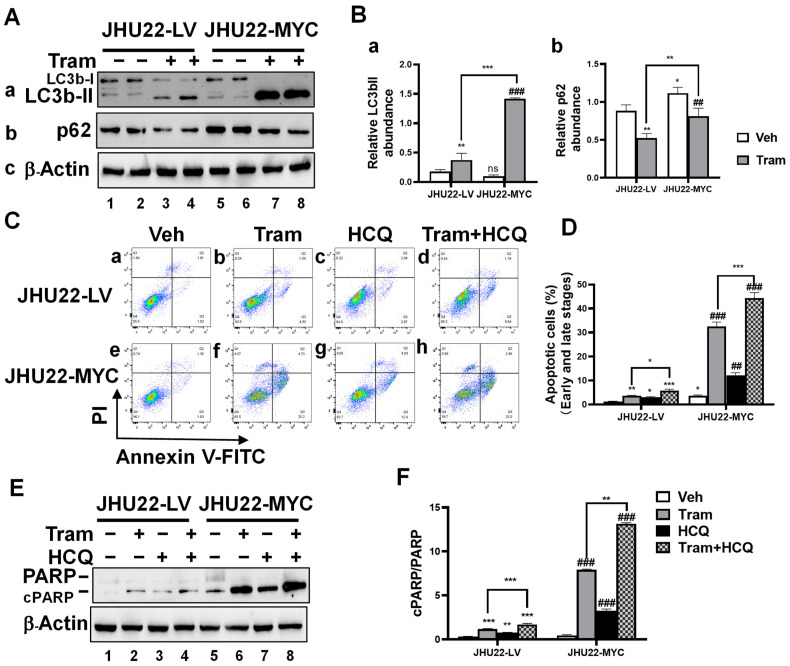
Inhibition of trametinib-induced autophagy enhances apoptotic cell death in HNSCC cells. (**A**) Total protein extracts were prepared from JHU22-LV and JHU22-MYC cells treated with either vehicle or trametinib (200 nM) for 48 h. Western blot analysis was performed to detect LC3bI/II (**a**), p62 (**b**), and β-actin (**c**). (**B**) Protein band intensities of LC3bII (**a**) and p62 (**b**) were quantified relative to the corresponding β-actin to compare the effects between vehicle-treated and trametinib-treated cells (n = 4). (**C**) JHU22-LV and JHU22-MYC cells were treated with vehicle (**a**,**e**), trametinib (**b**,**f**; 200 nM), HCQ (**c**,**g**; 10 µM), or the combination of trametinib and HCQ (**d**,**h**) for 48 h, then collected for annexin V and PI staining and analyzed via flow cytometry to assess early and late-stage apoptosis. (**D**) The populations of apoptotic cells in both early and late stages were quantified (n = 4). (**E**) Total protein extracts were prepared from JHU22-LV and JHU22-MYC cells treated with either vehicle, trametinib (200 nM), HCQ (10 µM), or the combination of trametinib and HCQ for 48 h. Western blot analysis was performed to detect cPARP/PARP and β-actin. (**F**) The ratio of cPARP/PARP was quantified to compare the effects between vehicle-treated and trametinib-treated cells. Data from four independent experiments are presented as mean ± SD (n = 4). Statistical significance is indicated as follows: * *p* < 0.05; ** *p* < 0.01; *** *p* < 0.001 compared to JHU22-LV vehicle-treated controls. ## *p* < 0.01; ### *p* < 0.001 compared to JHU22-MYC vehicle-treated controls. ns: not significant. Abbreviations: Veh: Vehicle, Tram: Trametinib, HCQ: hydroxychloroquine.

Autophagy is a cellular mechanism that provides energy and facilitates the clearance of misfolded or aggregated proteins. LC3bI, present in the cytoplasm, is converted into LC3bII through conjugation with the lipid phosphatidylethanolamine, which associates with the autophagosome membrane and serves as a marker of autophagy. The protein p62, a substrate for autophagic degradation, shows decreased levels as an indicator of autophagic clearance [29]. While trametinib effectively inhibits MEK signaling, reducing cell proliferation and increasing apoptosis in HNSCC cells, autophagy activation has emerged as a potential resistance mechanism that may limit its effectiveness. To examine whether autophagy plays a role in MYC-induced changes in trametinib’s effects in HNSCC, JHU22-LV and JHU22-MYC cells were treated with either a vehicle or trametinib (200 nM) for 48 h, followed by a Western blot analysis to assess the expression levels of autophagy-related proteins LC3b and p62. Although MYC overexpression did not affect LC3bII (Figure 5(Aa), bands 5 and 6 vs. bands 1 and 2; Figure 5(Ba)) levels, it significantly increased p62 (Figure 5(Ab), bands 5 and 6 vs. bands 1 and 2; Figure 5(Bb)) levels in JHU22-MYC compared with JHU22-LV cells. This suggests that MYC overexpression may lead to decreased autophagy activity in JHU22-MYC relative to JHU22-LV cells. In contrast, trametinib treatment resulted in a significant increase in LC3bII levels in both JHU22-LV and JHU22-MYC cells, along with a reduction in p62 levels compared to their respective vehicle-treated controls, suggesting trametinib induced significant autophagic activity in both cell types. Notably, these changes were more pronounced in JHU22-MYC cells compared to JHU22-LV cells (Figure 5(Aa,Ba) for LC3bII and Figure 5(Ab,Bb) for p62, bands 7 and 8 vs. bands 3 and 4), indicating that trametinib may release the suppressed autophagy activity by MYC overexpression.

To investigate whether inhibiting autophagy affects trametinib-induced apoptosis in HNSCC concerning MYC overexpression, we examined the effects of combining trametinib with the autophagy inhibitor hydroxychloroquine (HCQ). Cells were treated with a vehicle, trametinib (200 nM), HCQ (10 μM), or a combination of trametinib and HCQ for 48 h. MYC overexpression consistently increased apoptosis (both early and late stages) in JHU22-MYC compared to JHU22-LV cells (Figure 5(Ce) vs. Figure 5(Ca); Figure 5D). Treatment with trametinib (Figure 5(Cb) for JHU22-LV; Figure 5(Cf) for JHU22-MYC) and HCQ (Figure 5(Cc) for JHU22-LV; Figure 5(Cg) for JHU22-MYC) alone induced significant apoptosis in both cell lines compared to their respective vehicle controls. Notably, the combination of trametinib and HCQ (Figure 5(Cd) for JHU22-LV; Figure 5(Ch) for JHU22-MYC) significantly increased apoptosis compared to either vehicle or single-agent treatments, with the highest percentage of apoptotic cells observed in MYC-overexpressing cells. This was further confirmed by a Western blot analysis of the highest cPARP/PARP ratio that occurred in JHU22-MYC cells undergoing the combination treatment (Figure 5E, band 8; Figure 5F), indicating that combining trametinib and HCQ can enhance apoptosis, particularly in MYC-overexpressing cells.

In summary, our findings suggest that trametinib treatment induced an autophagy response in HNSCC, which may act as a resistance mechanism. The inhibition of autophagy with HCQ enhanced apoptosis, particularly in HNSCC cells with MYC overexpression. This indicates that targeting autophagy with HCQ could improve the therapeutic efficacy of trametinib in treating cancers characterized by elevated MYC expression.

### 2.6. Impact of Trametinib on Xenograft Tumors Overexpressing MYC in HNSCC

**Figure 6:**
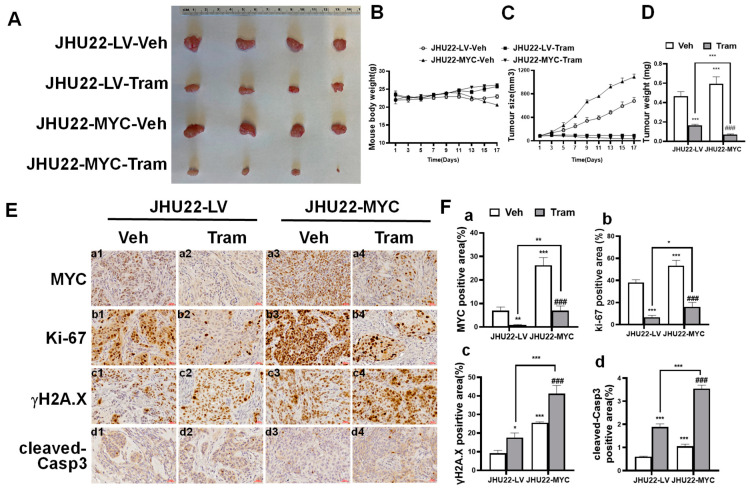
Impact of trametinib on xenograft tumors overexpressing MYC in HNSCC. JHU22-LV and JHU22-MYC cells were injected subcutaneously into athymic nude mice to form xenograft tumors (n = 4). (**A**) Representative images of the gross tumors. (**B**) Average body weights of the mice were monitored during tumor development. (**C**) Tumor sizes were measured every 2–3 days following injection and calculated using the formula: length × width^2^ × 0.5. (**D**) Comparison of the average weights of dissected tumors from mice. (**E**) IHC images showing MYC (JHU22-LV: a1, a2; JHU22-MYC: a3, a4), Ki-67 (JHU22-LV: b1, b2; JHU22-MYC: b3, b4), γH2A.X (JHU22-LV: c1, c2; JHU22-MYC: c3, c4), and cleaved-Casp3 (JHU22-LV: d1, d2; JHU22-MYC: d3, d4) in the HNSCC xenograft tumors treated with Veh or Tram, respectively. Scale bar = 30 µm (insets). (**F**) Quantification of the IHC staining for MYC (**a**), Ki-67 (**b**), γH2A.X (**c**), and cleaved-Caspase 3 (**d**). Data from four independent experiments are presented as mean ± SD (n = 4). Statistical significance is indicated as follows: * *p* < 0.05; ** *p* < 0.01; *** *p* < 0.001 compared to JHU22-LV vehicle-treated controls. ### *p* < 0.001 compared to JHU22-MYC vehicle-treated controls. Abbreviations: Veh: Vehicle, Tram: Trametinib, cleaved-Casp3: cleaved-Caspase 3.

To evaluate the therapeutic potential of trametinib and assess the impact of MYC overexpression in a more clinically relevant model, we examined its effects on xenograft tumors with MYC overexpression in mice. JHU22-LV and JHU22-MYC cells were inoculated into the right flank of nude mice (n = 4 per group, with the experiment repeated twice). The mice were subsequently treated with either a vehicle or trametinib when the tumors reached approximately 80 mm^3^. Photographs of the excised tumors revealed that trametinib-treated tumors, particularly those with MYC overexpression, were significantly smaller than those from vehicle-treated mice (Figure 6A). Body weights were monitored every 2–3 days over the treatment period during the experiment, and the body weight of each group remained largely stable. However, there was a notable drop in the body weight of JHU22-MYC vehicle-treated mice, which may be associated with the larger tumor size observed in this group. In contrast, the body weight of mice in the trametinib-treated groups showed no significant drop, indicating that trametinib did not negatively affect the overall health (Figure 6B). Tumor volumes were measured every 2–3 days post-injection, and the results showed that trametinib significantly inhibited tumor growth, reducing the tumor size by 30% in JHU22-LV tumors and by 50.2% in JHU22-MYC tumors compared to their respective vehicle-treated controls at the study endpoint. A more pronounced effect was observed in MYC-overexpressing tumors (Figure 6C). At the point of tumor excision, MYC-overexpressing tumors from trametinib-treated mice were significantly lighter than those from both vehicle-treated and trametinib-treated control mice. The tumor weight was highest in JHU22-MYC vehicle-treated mice and significantly decreased after trametinib treatment, remaining lower than in all other groups, including JHU22-LV trametinib-treated tumors (Figure 6D).

To investigate whether the molecular alterations observed in vitro could be recapitulated in vivo, we assessed the expression of key regulators of cell proliferation, DNA damage, and apoptosis on tumor sections. IHC staining revealed significantly elevated MYC protein levels in JHU22-MYC tumors compared to JHU22-LV tumors (Figure 6(Ea3) vs. Figure 6(Ea1); Figure 6Fa). After trametinib treatment, MYC expression was notably downregulated, with levels decreasing from 6.9% to 0.8% in JHU22-LV tumors (Figure 6(Ea1) vs. Figure 6(Ea2); Figure 6(Fa)) and from 26.3% to 7.0% in JHU22-MYC tumors (Figure 6(Ea3) vs. Figure 6(Ea4); Figure 6(Fa)). This suggests that while trametinib effectively reduced MYC expression in both tumor types, JHU22-MYC tumors retained a higher residual level of MYC-expressing cells after treatment. Additionally, trametinib reduced the expression of the proliferation marker Ki-67 in both tumor groups, though residual Ki-67 levels remained higher in JHU22-MYC tumors, consistent with their elevated MYC expression. Ki-67 levels decreased from 53.3% to 16.0% (Figure 6(Eb4) vs. Figure 6(Eb3); Figure 6Fb) compared to a reduction from 38.2% to 6.6% in JHU22-LV tumors (Figure 6(Eb2) vs. Figure 6(Eb1); Figure 6(Fb)). Further analysis showed that trametinib treatment led to a significantly greater increase in the expression of DNA damage marker γH2A.X, which increased from 25.6% to 41.3% in JHU22-MYC tumors (Figure 6(Ec3) vs. Figure 6(Ec4); Figure 6(Fc)) compared to the increase from 9.3% to 17.6% in JHU22-LV tumors (Figure 6(Ec1) vs. Figure 6(Ec2); Figure 6(Fc)). Similarly, cleaved-Caspase3, a marker of apoptosis, was significantly elevated in JHU22-MYC tumors, increasing from 1.1% to 3.5% (Figure 6(Ed3) vs. Figure 6(Ed4); Figure 6(Fd)), compared to an increase from 0.6% to 1.9% in JHU22-LV tumors (Figure 6(Ed1) vs. Figure 6(Ed2); Figure 6Fd) after trametinib treatment. These results demonstrate that trametinib effectively inhibited tumor growth, enhanced the DNA damage response, and induced apoptosis in both JHU22-LV and JHU22-MYC xenograft tumors, with more pronounced effects in MYC-overexpressing tumors. The prominent molecular alterations observed in JHU22-MYC tumors following trametinib treatment, which mirrored those seen in vitro, likely contributed to the greater inhibition of tumor growth compared to JHU22-LV tumors.

## 3. Discussion

Identifying novel therapeutic biomarkers and strategies for HNSCC remains a significant clinical challenge. Building on our previous findings, where MEK inhibition via trametinib demonstrated efficacy in treating HNSCC [17], this study extended its investigation to include not only the molecular alterations underlying these effects but also the role of oncogenic MYC in modulating HNSCC vulnerability to MEK inhibition with trametinib in in vitro and in vivo settings. Our results demonstrated that MYC overexpression sensitized HNSCC cells to the anti-tumor effects of trametinib, as evidenced by the lower IC50 value in JHU22-MYC cells compared to JHU22-LV cells. Functionally, MYC overexpression further delayed wound healing and colony formation, processes already inhibited by trametinib. In vivo xenograft models supported these findings, showing heightened sensitivity to trametinib in MYC-overexpressing tumors. Additionally, we found that inhibiting autophagy potentiated apoptotic cell death in HNSCC cells in response to trametinib, suggesting that autophagy may act as a resistance mechanism and that its suppression could enhance the efficacy of trametinib.

Up to 90% of HNSCC tumors overexpress EGFR [30], activating key intracellular signaling pathways such as MEK/ERK and PI3K/AKT, which promote cellular proliferation, survival, invasion, and metastasis [31,32]. Clinical studies have demonstrated the efficacy of EGFR inhibitors, such as monoclonal antibodies and tyrosine kinase inhibitors, in enhancing the effects of radiation and chemotherapy in HNSCC [33]. Our group’s work on recombinant immunotoxins targeting overexpressed EGFR has also shown anti-tumor efficacy in HNSCC [34,35]. However, the FDA-approved anti-EGFR antibody cetuximab, when used as monotherapy, has achieved only a 10–30% response rate, with resistance often emerging. EGFR knockdown reduces ERK activity with limited impact on AKT and mTOR signaling [36], emphasizing the importance of the EGFR-MAPK axis in HNSCC. Activation of kinases such as BRAF, KRAS, and ERK1/2 within the MAPK pathway is known to drive tumorigenesis and promote invasion [37]. MEK inhibition has been shown to reduce Ras/MEK/ERK pathway activation, with clinical trials in oral cavity HNSCC patients demonstrating promising tumor responses [17]. Prolonged cetuximab administration has been reported to induce a rebound in ERK1/2 phosphorylation, contributing to the treatment resistance. Elevated levels of pERK1/2 and pEGFR are potential risk factors for poor outcomes in HNSCC patients [38], reinforcing the need to target the EGFR-MAPK in therapeutic strategies. Our current research showed that MEK inhibition by trametinib not only suppressed EGFR activation but also induced molecular alterations related to DNA-damaged responses, transcription activation inhibition, cell cycle arrest, and apoptosis. These molecular alterations correspond to the observed cell proliferation suppression and enhanced cell death in response to trametinib in HNSCC. Specifically, trametinib treatment resulted in a marked G1 phase arrest, reduced G2/S phase progression, and significantly increased apoptosis.

The MYC transcription factor is one of the most potent and frequently deregulated oncoproteins in human cancers [39]. In this study, MYC overexpression in HNSCC cells accelerated cell cycle progression and proliferation while promoting DNA damage marker expression, consistent with its known role in cell transformation [40]. As a key mediator in the MAPK pathway, MYC expression was significantly reduced by trametinib, indicating MYC functions downstream of MEK signaling in these cells. Importantly, trametinib treatment alone induced DNA damage, and MYC overexpression further amplified this effect, leading to increased DNA damage accumulation in HNSCC cells. This was accompanied by elevated levels of pro-apoptotic proteins, including BIM, p53, and cleaved PARP, suggesting that these factors contribute to MYC-enhanced sensitivity to trametinib-induced apoptosis. The reduction in the G1 phase arrest and lower levels of p27 and p21 after trametinib treatment highlight the increased proliferation and metabolic activity driven by MYC overexpression. This rapid cell cycle progression, combined with diminished cell cycle inhibition, may render MYC-overexpressing cells more susceptible to apoptotic signals. Inhibition of MEK signaling by trametinib likely disrupts critical survival pathways, tipping the balance towards apoptosis in these cells. This process may reflect oncogene addiction [40], where cancer cells become heavily reliant on continuous MYC activity for survival and proliferation. Indeed, our data on the heightened sensitivity of JHU22-MYC cells compared to JHU22-LV cells in response to both trametinib and erlotinib may support this notion. Thus, targeting pathways that sustain MYC activity, particularly in the context of MEK inhibition, presents a promising therapeutic strategy to exploit this addiction and enhance treatment efficacy in HNSCC.

The relationship between MYC expression levels and treatment sensitivity in cancer is conflicting. While some studies suggest that MYC overexpression leads to drug resistance [24,25,41,42,43], others—including our own [17]—show that MYC can enhance sensitivity to certain therapies. For example, MYC-overexpressing cells are more sensitive to mitotic disruptions and inhibitors of Aurora kinases and CDK1 [44,45,46]. MYC overexpression also increases sensitivity to chemotherapeutic agents targeting microtubules, topoisomerases, and DNA/RNA/protein synthesis, making these cells more vulnerable to mitotic arrest and cell death [47,48]. Additionally, MYC hyperactivation in pancreatic cancer is linked to increased sensitivity to SUMO inhibition [49]. In HNSCC, MYC overexpression correlates with poor prognosis, and its inhibition reduces proliferation, invasion, and migration [23]. Here, we demonstrated that MYC overexpression enhanced HNSCC vulnerability to MEK inhibition, highlighting the potential for targeting MYC in HNSCC therapy. In MYC-overexpressing HNSCC cells, we identified a dependence on the MEK/ERK pathway for survival, suggesting that MYC could be a predictive marker for treatment responses. Future studies are needed to explore the molecular mechanisms behind these enhanced drug responses.

We found that dual inhibition of autophagy and MEK signaling significantly impacted HNSCC cells. Autophagy inhibition is a known strategy in cancer therapy; for instance, in B-cell malignancies, it helps overcome resistance to CAR-T immunotherapy [50]. Single MEK or HSP90 inhibition induces autophagy (marked by LC3b), and this effect is amplified with dual inhibition, suggesting HSP90 inhibition does not sensitize pancreatic cancer to MEK inhibition via autophagy suppression [51]. The dual inhibition of autophagy and MEK [52] has also been shown to promote ferroptosis in Lkb1-deficient Kras-driven lung tumors, with HCQ and trametinib displaying synergistic anti-proliferative activity. In lung cancer [53,54,55], autophagy contributes to resistance to tyrosine kinase inhibitors (TKIs), and autophagy inhibition increases TKI sensitivity via apoptosis. Additionally, PD-L1 upregulation induces autophagy through the MAPK pathway, promoting tumor progression and resistance to EGFR inhibitors [56]. While the role of autophagy in HNSCC is still under investigation, it is regulated by the EGFR/MAPK and PI3K/Akt pathways [57]. In our study, trametinib induced autophagy, as evidenced by increased LC3bII and decreased p62 levels. Combining autophagy inhibition using HCQ and MEK inhibition via trametinib enhanced apoptosis in HNSCC cells, suggesting that trametinib-induced autophagy is protective and that there is a synthetic lethality between autophagy and MEK signaling. In MYC-overexpressing HNSCC cells, this dual inhibition elicited an even stronger apoptotic response. Therefore, combining MEK and autophagy inhibition may enhance the cytotoxicity of trametinib in HNSCC.

A limitation of the current study is its focus on tumor-cell-intrinsic MYC, specifically regarding its role downstream of the MEK/MAPK pathway in regulating processes such as cell survival, cell cycle progression, proliferation, DNA damage, and apoptotic cell death, as revealed by MEK inhibition with trametinib. While informative, this narrow focus may limit our broader understanding of MYC’s role in HNSCC and its interaction with other signaling pathways. Additionally, the use of MYC overexpression in our models prevented us from establishing a clear cut-off value for MYC expression in HNSCC cells, which could provide more precise insights into its role in tumor progression and treatment responses. Future studies should aim to expand beyond cell-intrinsic mechanisms by investigating MYC’s interactions with other key signaling pathways and the tumor microenvironment. Such research could provide a more comprehensive understanding of MYC’s broader impact on HNSCC. Additionally, establishing a threshold for MYC expression would enhance patient stratification, enabling the identification of those most likely to benefit from MEK inhibition or other targeted therapies. Investigating the molecular mechanisms driving the enhanced response to MEK inhibition in MYC-overexpressing cells could also lead to more personalized treatment strategies, optimizing outcomes for patients with MYC deregulation. Nonetheless, our data suggest that understanding these mechanisms is crucial for improving treatments for HNSCC tumors with MYC dysregulation.

## 4. Materials and Methods

### 4.1. Cell Cultures

The JHU-011 (JHU11), JHU-013 (JHU13), and JHU-022 (JHU22) HNSCC cell lines, kindly provided by John Hopkins University (Baltimore, MD, USA) [17,34,35], were cultured in media RPMI1640 (#11875-093, Gibco, Thermo Fisher Scientific, Waltham, MA, USA) and supplemented with 10% fetal bovine serum (FBS, #A5256701, Gibco) and a 1% antibiotic-antimycotic solution (#15140-122, Gibco). The cells were maintained in a humidified incubator at 37 °C with 5% CO_2_.

### 4.2. Exogenous Overexpression of MYC in the JHU22 HNSCC Cells

A negative control lentivirus and a MYC-expressing lentivirus were purchased from Genecopoeia (Rockville, MD, USA). JHU22 cells were transduced with the lentivirus at a multiplicity of infection (MOI) of 5, using 10 μg/mL polybrene (#TR-1003-G, Sigma-Aldrich, St. Louis, MO, USA) to enhance the infection. Cells successfully infected with either the control lentivirus (JHU22-LV) or the MYC-expressing lentivirus (JHU22-MYC) were selected over a 14-day period with 2.5 μg/mL puromycin (#A11138-03, Gibco). The selected cells were then plated in 6-well plates and treated with either a vehicle control or trametinib (#S2673, Selleck Chemicals, Houston, TX, USA). Afterward, the cells were harvested for cell cycle analysis, apoptosis assays, or Western blot analysis.

### 4.3. Cell Viability Assay

Cell viability was assessed using the Crystal Violet Assay Kit (#ab232855, Abcam, Cambridge, UK). JHU22-LV and JHU22-MYC cells were seeded in 96-well plates at a density of 3000 cells per well and cultured for 7 days. After treatment with either a vehicle or trametinib, following a 2-fold serial dilution, a crystal violet staining assay was performed to evaluate cytotoxicity and cell viability. Optical density (OD) was measured at 595 nm using a microplate reader. Cell viability was calculated as the percentage of viable (attached) cells compared to the vehicle-treated control samples as previously described [17,34].

### 4.4. Colony Formation Assay

For the colony formation assay, three hundred JHU22-LV or JHU22-MYC cells were seeded into each well of a 6-well plate. The cells were treated with either a vehicle or trametinib (200 nM) and cultured in a medium for 5–7 days, with the medium being replaced every 2 days. Afterward, the colonies were fixed with methanol and stained with 0.1% crystal violet (#C0775, Sigma-Aldrich) in PBS for 20 min. Colony formation was assessed by counting the percentage of positive areas of stained colonies by Image J (ImageJ, National Institutes of Health, Bethesda, MD, USA). Each group was evaluated in triplicate, and the photos were analyzed using Image J.

### 4.5. Wound Healing Assay

For the wound healing assay using a scratch experiment, a specific number of JHU22-LV and JHU22-MYC cells were seeded into 24-well plates. Once the cells reached approximately 80% confluence, a sterile pipette tip (Ø = 0.1 mm) was used to gently scratch a straight line across the center of the well. The wound area was photographed using an inverted microscope at 0 (before drug treatment) and again at 6 and 24 h after treatment with either a vehicle or trametinib (200 nM). Images were captured at 20× magnification, ensuring both scratch edges were visible. Wound closure was quantified using ImageJ software (ImageJ Fiji). The wound closure percentage was calculated based on the reduction in the wound area over time using the formula Wound Closure (%) = [A(0) − A(t)] ÷ A(0) × 100, where A(0) is the initial wound area and A(t) is the area at the specific time point.

### 4.6. Flow Cytometric Analysis of Apoptosis and Cell Cycle

For the cell apoptosis analysis, 3 × 10^5^ JHU22-LV and JHU22-MYC cells were seeded into each well of a 6-well plate. Once the cells reached approximately 30–40% confluence, they were treated with a vehicle, trametinib (200 nM), hydroxychloroquine (HCQ, 10 μM) (#S4430, Selleck Chemicals), or a combination of trametinib and HCQ for 48 h. Following treatment, cells were double-stained with fluorescein isothiocyanate (FITC)-Annexin V and propidium iodide (PI) using the FITC Annexin V Apoptosis Detection Kit (#556547, BD Biosciences, San Jose, CA, USA), according to the manufacturer’s instructions. Apoptotic and viable cells were analyzed using a flow Cytometer (#A00-1-1102, Beckman Coulter, Brea, CA, USA) equipped with CytExpert software (CytExpert 2.4), and then the data were analyzed by FlowJo10 (FlowJo, Ashland, OR USA). The proportion of apoptotic cells was determined and compared to the vehicle group.

For the cell cycle analysis, 3 × 10^5^ JHU22-LV and JHU22-MYC cells were seeded into each well of a 6-well plate one day prior to treatment. They were then treated with vehicle or 200 nM trametinib for 48 h. Following treatment, cells were stained with 4′,6-diamidino-2-phenylindole (DAPI; #62248, Thermo Fisher Scientific) and analyzed by a flow Cytometer (Beckman Coulter). The data were analyzed by FlowJo10, and the percentages of cells in the G1 and G2/M phases were quantified and compared across samples. All assays were conducted in triplicate.

### 4.7. Western Blot and Antibodies

Cell protein lysates were separated using 10% sodium dodecyl sulfate-polyacrylamide gel electrophoresis (SDS-PAGE), transferred onto 0.2 μm nitrocellulose (NC) membranes (#1704158, Bio-Rad Laboratories, Hercules, CA, USA), and incubated with specific antibodies. Protein band intensities were analyzed by densitometry using Quantity One software (ChemiDoc^TM^ MP Imaging System, Serial No. 734BR2048, Bio-Rad). β-Actin was used as a loading control. The antibodies used in this study were sourced as follows: pEGFR (#2234S), pERK1/2 (#9101S), ERK1/2 (#9102S), γH2A.X (#7631S), pSer7 (#13780S), pSer2/5 (#4735S), PARP (#9542S), BIM (#2933S), p27 (#3686S), p62 (#5114S), and LC3b (#2775S) were obtained from Cell Signaling Technology (CST, Danvers, MA, USA). Additionally, antibodies for E2F1 (#SAB4500682), p53 (#P6749), and p21 (#P1484) were purchased from Sigma, while MYC (#ab32072) was acquired from Abcam. Immunoblotting was carried out using HRP-conjugated secondary antibodies, and specific protein bands were visualized with an enhanced chemiluminescence (ECL) (#1705062, Bio-Rad) detection system. Protein band intensities were quantified using ImageJ software.

### 4.8. Xenograft Tumor Models in Athymic Nude Mice

To validate our in vitro findings, we conducted in vivo studies in compliance with ethical standards. All experimental procedures were approved by the Howard University Animal Care and Use Committee, following the NIH Guide for the Care and Use of Laboratory Animals. Five- to six-week-old male athymic NU/NU nude mice (Charles River Laboratories, Frederick, MD, USA) were used for xenograft models. A total of 5 million JHU22-LV or JHU22-MYC cells, suspended in 100 μL of a medium containing 50% Matrigel basement membrane matrix (#354234, BD Biosciences), were subcutaneously injected into the right flank of the mice. Once the median tumor size reached approximately 80 mm^3^, the mice were randomized into treatment groups to receive either a vehicle or trametinib (1 mg/kg/day) treatment via oral gavage, administered once daily for 5 days each week over a 17-day treatment period. Tumor sizes were measured with calipers every 2–3 days, and body weight was monitored to assess overall health. At the end of the treatment period, the mice were euthanized, and tumors were harvested for further analysis. Statistical evaluations included a two-way ANOVA for tumor growth analysis and a one-way ANOVA for assessing differences in tumor weight.

### 4.9. Immunohistochemical (IHC) Analysis

IHC staining was performed using the avidin–biotin–peroxidase complex method, as previously described [34,35]. Paraffin-embedded tissue sections were first dewaxed and rehydrated. To block endogenous peroxidase activity, the sections were treated with 30% hydrogen peroxide, followed by rinsing with PBS (pH 7.6). The slides were then incubated overnight at 4 °C with primary antibodies targeting γH2A.X (Ser139) (#80312S, Cell Signaling Technology), cleaved caspase3 (#9664S, Cell Signaling Technology), Ki-67 (#9027S, Cell Signaling Technology), and MYC (#ab32072, Abcam). After rinsing with PBS for 5 min, repeated 5 times, the sections were incubated with secondary antibodies (biotinylated goat anti-rabbit IgG and goat anti-mouse IgG, Sigma). Following an additional wash for 5 min, repeated 5 times, the tissues were incubated with DAB (#SK-4100, Vector Laboratories, Burlingame, CA, USA) for 1–5 min, and the sections were counterstained with Mayer’s hematoxylin.

### 4.10. Statistics

All data are presented as the mean ± standard deviation (SD). Comparisons between two groups were analyzed using a two-tailed Student’s t-test, while comparisons among multiple groups were performed using a one-way ANOVA or two-way ANOVA for comparing different groups, followed by post hoc comparisons using Tukey’s multiple comparison test. Survival curves were compared using the log–rank (Mantel–Cox) test, and statistical significance was determined based on the chi-square distribution. GraphPad Prism 8 software was employed for statistical analysis. All tests were two-sided, and a *p*-value of less than 0.05 was considered statistically significant.

## 5. Conclusions

In conclusion, this research demonstrated a synergistic interaction between MYC-driven cell cycle progression and trametinib-induced MEK inhibition, enhancing HNSCC sensitivity to DNA damage and apoptotic cell death. Additionally, autophagy inhibition may potentiate trametinib’s efficacy. Our findings highlight MEK/MAPK signaling as a promising therapeutic target in HNSCC, particularly when combined with autophagy inhibition.

## Data Availability

All data will be made available upon request.

## Supplementary Materials

The following supporting information can be downloaded at https://www.mdpi.com/article/10.3390/ijms26020588/s1.
