# Supplementary material for: MYC Overexpression Enhances Sensitivity to MEK Inhibition in Head and Neck Squamous Cell Carcinoma
Source: Int J Mol Sci. 2025 Jan 12;26(2):588. doi: 10.3390/ijms26020588 (PMC11766173; doi:10.3390/ijms26020588)
Supplement: Supplementary file 1 [file ijms-26-00588-s001.zip › ijms-3390077-supplementary.pdf]

Article

MYC Overexpression Enhances Sensitivity to MEK Inhibition in Head and Neck Squamous Cell Carcinoma

Cuicui Yang [1,2], Xiaowu Pang [1], Shaolei Teng [3], Shamel Wilson [1], Xinbin Gu [1,2] and Guiqin Xie [1,2] *

[1] Department of Oral Pathology, Howard University, 600 W Street NW, Washington, DC 20059, USA; cuicui.yang@howard.edu (C.Y); xpang@howard.edu (X.P); shamel.wilson@bison.howard.edu (S.W)
[2] Cancer Center, Howard University, 2041 Georgia Avenue NW, Washington, DC 20059, USA; xgu@howard.edu (X.G)
[3] Department of Biology, Howard University, 415 College St. NW, Washington, DC 20059, USA; shaolei.teng@howard.edu (S.T)
* Correspondence: guiqin.xie@howard.edu (G.X)

Suppl Figure 1:

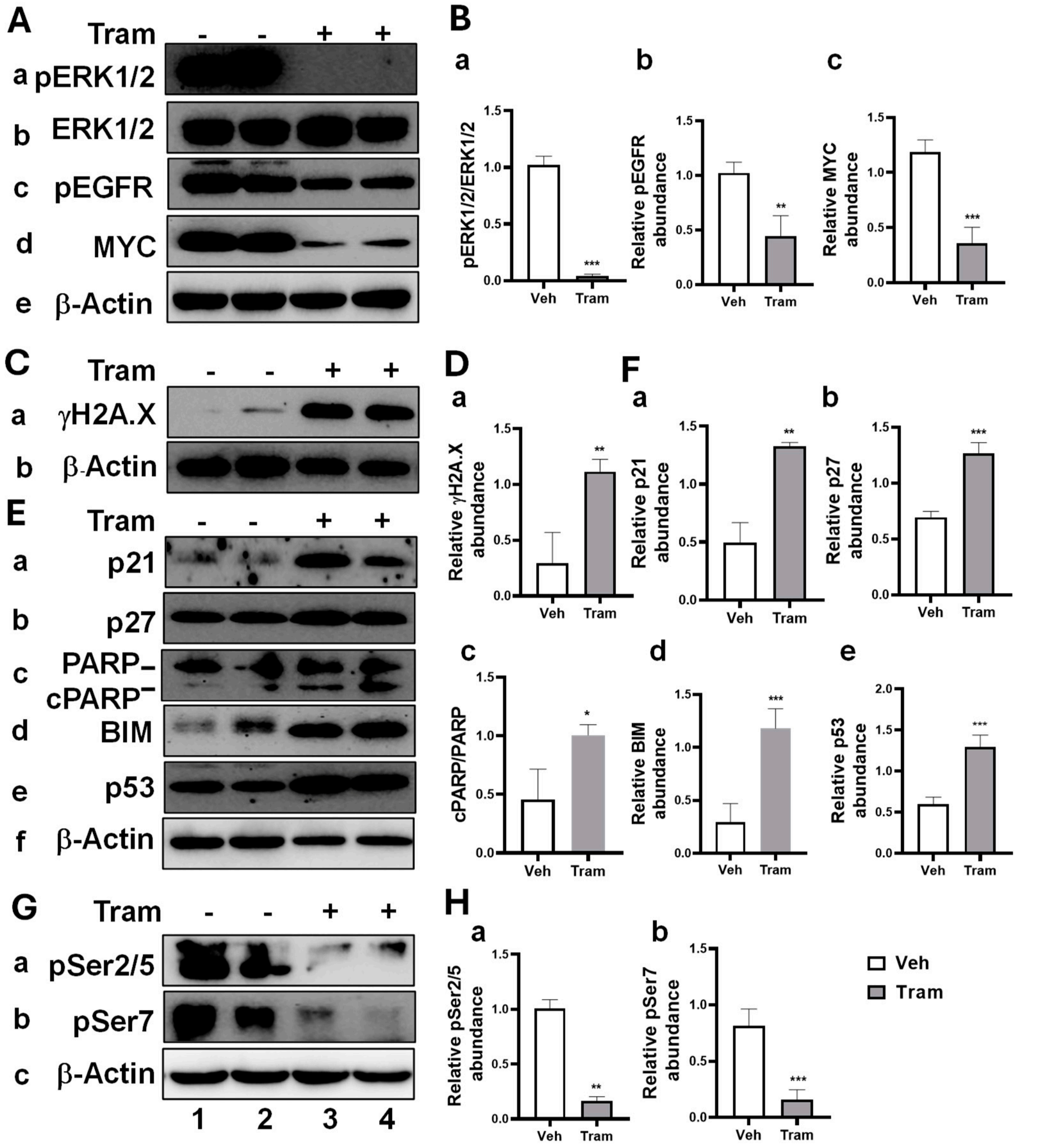

Suppl Figure 1. Multiple anti-cancer effects induced by trametinib through MEK/MAPK inhibition in JHU13 HNSCC cells. Total protein extracts were prepared from JHU13 cells treated with either vehicle or 200 nM trametinib for 48 hours. Western blot analysis was performed to detect: (A) pERK1/2 (a), total ERK1/2 (b), pEGFR (c), and MYC (d). (B) The ratio of (a) pERK/ERK and protein band intensities of (b) pEGFR as well as (c) MYC were quantified relative to the corresponding β-actin to compare vehicle- and trametinib-treated cells. (C-D) γH2A.X (a); (E-F) p21 (a), p27 (b), cPARP/PARP (c), BIM (d), and p53 (e); (G-H) pSer2/5 (a) and pSer7 (b). The ratio of cPARP/PARP and protein band intensities for pEGFR, MYC, γH2A.X, p21, p27, BIM, p53, pSer2/5, and pSer7 were normalized to β-actin correspondingly and compared between vehicle-treated and trametinib-treated cells. Data from independent experiments are presented as mean ± SD (n =3 ~ 4). Statistical significance is indicated as follows: $*p < 0.05$, $**p < 0.01$, $***p < 0.001$ compared to vehicle-treated controls. Abbreviations: Veh: Vehicle, Tram: Trametinib.

Suppl Figure 2:

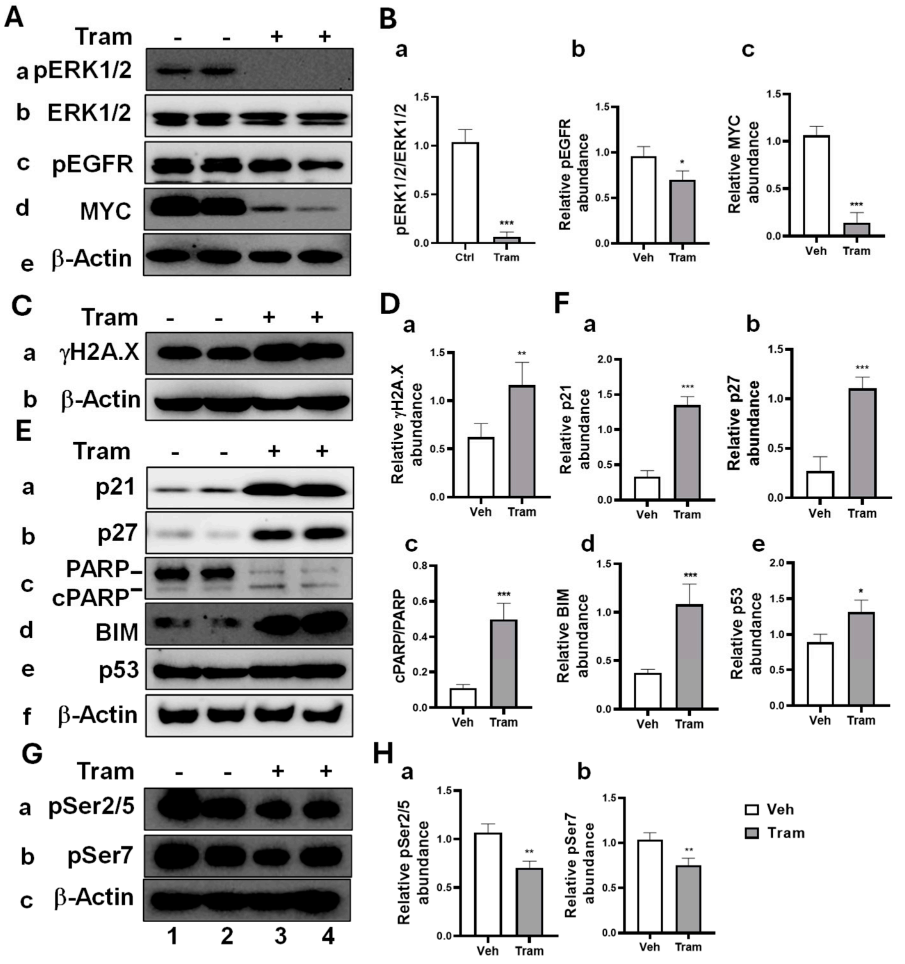

Suppl Figure 2. Multiple anti-cancer effects induced by trametinib through MEK/MAPK inhibition in JHU22 HNSCC cells. Total protein extracts were prepared from JHU22 cells treated with either vehicle or 200 nM trametinib for 48 hours. Western blot analysis was performed to detect: (A) pERK1/2 (a), total ERK1/2 (b), pEGFR (c), and MYC (d). (B) The ratio of pERK/ERK (a) and protein band intensities of pEGFR (b) as well as MYC (c) were quantified relative to the corresponding β-Actin to compare vehicle- and trametinib-treated cells. (C-D) γH2A.X (a); (E-F) p21 (a), p27 (b), cPARP/PARP (c), BIM (d), and p53 (e); (G-H) pSer2/5 (a) and pSer7 (b). The ratio of cPARP/PARP and protein band intensities for, pEGFR, MYC, γH2A.X, p21, p27, BIM, p53, pSer2/5, and pSer7 were normalized to β-actin correspondingly and compared between vehicle-treated and trametinib-treated cells. Data from independent experiments are presented as mean ± SD (n =3 ~ 4). Statistical significance is indicated as follows: **p* < 0.05, ***p* < 0.01, ****p* < 0.001 compared to vehicle-treated controls. Abbreviations: Veh: Vehicle, Tram: Trametinib.
